# Mental Imagery in Social Anxiety in Children and Young People: A Systematic Review

**DOI:** 10.1007/s10567-020-00316-2

**Published:** 2020-04-15

**Authors:** Jennifer Chapman, Brynjar Halldorsson, Cathy Creswell

**Affiliations:** 1 Oxford Health NHS Foundation Trust, University of Oxford, Oxford, UK; 2 Department of Psychiatry, Warneford Hospital, University of Oxford, Oxford, UK; 3 Department of Experimental Psychology, University of Oxford, Oxford, UK; 4 Department of Psychology, Reykjavik University, Reykjavík, Iceland

**Keywords:** Social anxiety, Imagery, Children and young people

## Abstract

Current cognitive models of social anxiety disorder (SAD) in adults indicate that negative self-images play a pivotal role in maintaining the disorder. However, little is known about the role of negative imagery in the maintenance of social anxiety for children and young people. We systematically reviewed studies that have investigated the association between imagery and social anxiety in children and young people. Four databases were searched for ‘social anxiety’ and related terms (including ‘social phobia’ and ‘performance anxiety’) combined with ‘imagery’, ‘representation*’, and ‘observer perspective’. The nine studies that met the inclusion criteria provided some evidence that children and young people with higher social anxiety report more negative, observer’s perspective images, and some evidence to support the cognitive models of SAD’s conceptualisation of imagery. Only two studies included samples with pre-adolescent children. The literature is limited by a number of methodological issues, including inconsistencies in, and a lack of good psychometric measures for, imagery in children and young people. More conclusive evidence is needed to develop significant and robust conclusions.

**Electronic supplementary material:**

The online version of this article (10.1007/s10567-020-00316-2) contains supplementary material, which is available to authorized users.

## Introduction

Social anxiety disorder (SAD) is one of the most common mental health problems (Kessler et al. 2005a, b), has an early age of onset (median age = 13 years; Kessler et al. 2005a, b) and an estimated prevalence in children and young people of between 3 and 10% (Merikangas et al. 2010; Wittchen and Fehm 2003). Full remission from SAD is uncommon without treatment (Bittner et al. 2007), and the presence of SAD during childhood and adolescence presents a risk for further mental health problems (e.g. depression and substance abuse) as an adult (Stein and Stein 2008). These considerations highlight the importance of effective, early intervention. However, a number of recent studies have suggested that children and young people with SAD have poorer outcomes than children with other anxiety disorders from multi-anxiety disorder-focused Cognitive Behavioural Therapy (CBT; Hudson et al. 2015), and that many children and young people continue to experience difficulties after SAD-specific treatments (Kodal et al. 2018). To improve treatment outcomes, it will be important to better develop an understanding of the SAD-specific maintenance processes that should be targeted to optimise treatment outcomes for children and young people with SAD (Halldorsson and Creswell 2017).

Cognitive models of SAD (Clark and Wells 1995; Rapee and Heimberg 1997) have provided a theoretical framework to understand the maintenance of this disorder in adults. In addition to interpretation biases, safety-seeking behaviours, and pre- and post-event processing, the models propose that negative imagery play a key role in maintaining the disorder. More specifically, the models suggest that when socially anxious individuals enter social situations they may experience spontaneously occurring images as if seen from an observer’s (rather than field) perspective (Clark 2005). These images are typically distorted and excessively negative, and often consist of the individual’s fears visualised. For example, a person afraid of blushing might have an image of themselves looking red and use this image to infer how they appear to others. In an effort to prevent or minimise the feared catastrophe, the cognitive models hypothesise that the individual may engage in safety-seeking behaviours (e.g. try to cover their face to prevent others from noticing blushing) and/or avoidance—which contribute to keeping the problem going (Clark 2005).

Consistent with the cognitive models, in a recent systematic review, Ng et al. (2014) found that social anxiety (symptoms or disorder) among adults was significantly associated with images that were from an observer’s perspective and were negative in valence, but was not associated with other imagery dimensions, such as vividness or duration of the negative image. Furthermore, the findings from experimental studies indicated that inducing negative imagery resulted in negative thoughts, and poorer observer-rated performance for socially anxious participants, as well as heightened anxiety and poorer self-rated performance for both socially anxious and non-anxious control participants.

Despite the strong support for the role of imagery in adult social anxiety, little is known about the role or nature of imagery in children and young people with social anxiety symptoms or SAD. Specifically, it is unclear whether a similar association exists between social anxiety and imagery across the age range, given the marked changes in cognitive abilities and structures through childhood and adolescence (Yurgelun-Todd 2007) in which children develop the ability to see oneself through the eyes of another, as well as developing a greater capacity for social comparisons (Cole et al. 2001). Given the central role of imagery in the treatment of adult social anxiety disorder, it is critical to understand whether and how imagery contributes to social anxiety in children and young people, in order to potentially inform the development of more effective treatments.

This systematic review aims to identify research that has investigated the association between imagery and social anxiety in children and young people. As there have been no prior reviews, this paper will synthesise and critically evaluate the broad literature in this area, replicating aspects of the approach taken in the recent review of adult studies (Ng et al. 2014) in order to examine the following: (i) the association between imagery characteristics and social anxiety (or SAD) experienced by children and young people and (ii) the effect/s of negative imagery on children and young people with high social anxiety (or SAD) across a range of outcome variables.

## Method

### Inclusion Criteria

(i) Paper was written in English;
(ii) Paper was published in a peer reviewed journal;
(iii) The paper reported on clinical or non-clinical samples of children or young people up to the age of 25. The 0–24 age group was chosen to align with recent conceptualisations of adolescence (Sawyer et al. 2018). Where the upper age limit of a sample exceeded 24 years, the study was included if subgroup analyses of children and young people (24 years and younger) were presented. If the mean age of participants was reported without the range, the age was determined using an assumption of normality that the sample mean age plus 3 standard deviations equalled less than 25;
(iv) There was a measure of social anxiety symptoms or diagnosis;
(v) There was a measure of the experience of imagery;
(vi) It was possible to extract data for an association between social anxiety and imagery.

### Exclusion Criteria

(i) Social anxiety was examined in the context of other comorbid conditions non-anxiety and/or non-mood disorders (e.g. psychosis, autism);
(ii) Social anxiety was measured in the context of physical health conditions/experiences (e.g. pain or surgery);
(iii) The paper did not report novel findings (e.g. was a review or meta-analysis);
(iv) The paper was published before Social Phobia was recognised in the diagnostic and statistical manual of mental disorders, third edition (DSM-III; American Psychiatric Association 1980)

### Search Strategy

The following databases were searched on 31st December 2018, and repeated on 19th January 2019: PsycInfo, CINAHL, MEDLINE, and EMBASE. Search terms included ‘social anxiety’ and related terms (such as, ‘social phobia’ and ‘performance anxiety’; following Mayo-Wilson et al. 2014) combined with ‘imagery’, ‘representation*’, or ‘observer perspective’ (see Supplementary material for full search terms). The first author also carried out a manual search on the reference lists of all articles meeting the inclusion criteria.

### Study Selection

**Fig. 1:**
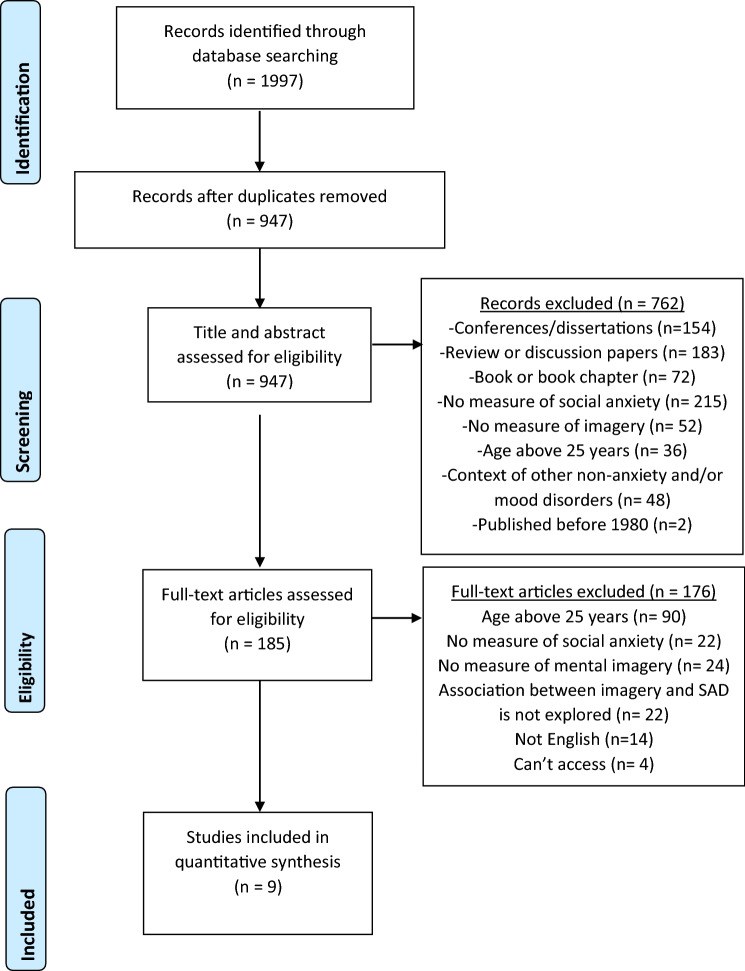
PRISMA flow chart of study selection process

A Preferred Reporting Items for Systematic Reviews and Meta-Analysis (PRISMA; Moher et al. 2009) flow chart (Fig. 1) describes the systematic review process. All titles and abstracts were screened for eligibility according to the inclusion and exclusion criteria above by two reviewers (JC and BH). If it was clear that the paper did not meet the criteria according to the title and abstract, then the paper did not advance to the next stage in which the full text was reviewed. There was moderate agreement between the two reviewers, *k* = 0.708 (95% CI 0.65 to 0.77), *p* < 0.001. Any papers included by either reviewer advanced to the next stage.

As shown in Fig. 1, the combined electronic database search retrieved 1997 records and 947 papers were retained after duplicates were removed. Seven hundred and sixty-two papers were excluded on the basis of the title and abstract not meeting the inclusion and exclusion criteria, and a further 176 papers failed to meet the criteria when the full texts were examined, leaving nine articles that met the inclusion criteria.

A second reviewer (BH) independently assessed all papers that advanced to the full-text examination stage. There was moderate agreement between the two reviewers, *k* = 0.692 (95% CI 0.49 to 0.89), *p* < 0.001. Any discrepancies in the inclusion of a paper were discussed between the two reviewers and a third reviewer was consulted (CC) in the case of any outstanding differences.

### Data Extraction and Quality Assessment

A data extraction tool was developed to promote reliability of data collection and reduce the risk of error. Data were extracted by the first reviewer, and included information, such as percentage of females, whether informed consent was obtained, and the imagery measures used.

The methodological quality of the included studies was assessed using a modified version of the quality rating checklist developed by Kmet et al. (2004), which covers basic elements of the design, such as whether the sample was adequately powered, or the measures employed were described in detail and had good psychometric properties. The wording of some items was adapted to match the current review, and non-relevant items (e.g. whether blinding of subjects was reported) were excluded. Items on the checklist were rated on a 3-point scale (0 = no, 1 = partial, 2 = yes) with a maximum score of 24. Two reviewers (JC and BH) independently assessed the quality of each included study with a high degree of reliability (intraclass correlation coefficient ((ICC) = 0.99 [95% CI 0.98 to 0.99]; *F*_(108)_ = 91.617, *p* < 0.001). Studies were classified into three groups on the basis of the average quality score across raters, low (0–14), medium (15–19), and high (20–24).

### Data Synthesis

Data were synthesised and organised using the following structure: (a) findings from studies that examine the association between imagery characteristics and social anxiety (or SAD); and (b) findings that examine the *effects* of negative imagery for individuals with high social anxiety (or SAD).

Social anxiety and imagery measures were assessed based on their observed psychometric properties. They were deemed to have good psychometric properties if they had been validated with the target population. Effect sizes (Cohen’s *d)* were included for all studies that reported them or where they could be were calculated (using https://www.psychometrica.de/effect_size.html). Effect sizes were interpreted using conventions proposed by Cohen (1988). An effect size of 0.2 was categorised as small, 0.5 as a medium effect, and 0.8 as a large effect size.

## Results

### Description of Included Studies

As shown in Table 1, the nine studies were published between 2005 and 2018 and included 1,496 participants. The mean reported participant age ranged from 10.2 to 19.7 years. Only two studies (Vassilopoulos and Moberly 2013; Vassilopoulos et al. 2012) included samples with pre-adolescent children (age 10–12 years). The percentage of female participants ranged from 50 to 80% across studies. The studies were all conducted in high-income countries, including United Kingdom, Greece, Japan, Finland, and Germany. Two research groups authored five of the nine studies (Schreiber et al. 2012; Schreiber and Steil 2013; Vassilopoulos 2005; Vassilopoulos and Moberly 2013; Vassilopoulos et al. 2012).

**Table 1:** Summary of included studies

Citation	Design	Participants (number and age)	Groups	Social anxiety measure	Imagery measure	Imagery induction/ manipulation	Other relevant measures	Mean quality rating (0–24)
Hignett and Cartwright-Hatton, (2008) UK	Correlational	*n* = 124 Age 12–18, *M* = 14.4 (SD = 0.46)	Students from local schools (age 12–14 and 16–18)	SPAI-C (SR) (+); FNE (SR) (+)	PTRS (SR) (?)	Completed PTRS and anxiety Likert scale following a speech task. Asked to imagine their speech performance	Anxiety Rating Likert Scale during speech task (SR) (−)	22
Moriya (2018) Japan	Correlational	*n* = 231 Age 18–23, *M* = 19.26 (SD = 0.83)	Undergraduates	BFNE (SR) (+)	VVIQ (SR) (?) VISQ (SR) (?) VVQ (SR) (?)	Asked to complete questionnaires with no imagery induction or manipulation	Effortful Control Scale (SR) (?)	18
Ranta et al. (2014) Finland	Between groups	*n* = 133 (306 in initial screening) *M* = 15.9 (SD = 0.32)	(1) HSA (*n* = 43) NSA (n = 90) (2) SAD (n = 10)/subclinical social anxiety (*n* = 7) No diagnosis (*n* = 116)	SPIN (SR) (+); interview with the K-SADS-PL (+)	Semi-structured interview (−)	Asked to imagine past anxious event	Semi-structured interview assessing most distressing situation, automatic thoughts, safety behaviours, and coping strategies (-)	21.5
Schreiber et al. (2012) Germany	Correlational (and between groups)	*n* = 567 14–20 years old, *M* = 16.49 (SD = 1.67)	HSA (*n* = 145) NSA (*n* = 143)	SPAI (SR) (+); Social Behaviour Questionnaire (SR) (?); Social Cognitions Questionnaire (SR) (?); Social Attitudes Questionnaire (SR) (?)	QRI-SP (SR) (−)	Asked to complete questionnaires with no imagery induction or manipulation	DICA (SR) (+);	22.5
Schreiber and Steil (2013) Germany	Between groups	*n* = 62 Age 14–20 SAD: M = 16.6 years (SD = 2.21) NAC: matched	SAD (*n* = 31) NSA (*n* = 31)	SPAI-C (SR) (+)	QRI-SP (SR) (?)	Asked to hold past negative image in mind while completing questionnaires No manipulation	DICA (SR) (+)	22
Stopa and Jenkins (2007) UK	Between groups	*n* = 20 M = 19 (SD = 1.07)	HSA (*n* = 20) Positive and negative imagery groups	FNE (SR) (+)	Vividness scale and ability to produce image in manipulation (SR) (−)	Asked to hold positive or negative image in mind during speech task	BDI-II (SR) (+), Likert scale for anxiety and performance (SR) (−) Behaviour checklist for speech performance (SR/CR) (−)	18.5
Vassilopoulos (2005) Greece	Between groups	*n* = 80 Low anxious: *M* = 19.67 (SD = 0.92) High anxious: *M* = 19.70 (SD = 0.69)	HSA (*n* = 40) NSA (*n* = 40) Positive and negative imagery groups	FNE (SR) (+); SPAI (SR) (+); STAI (SR) (+)	Self-imagery questionnaire (SR) (−)	High and low socially anxious were randomly allocated to positive and negative imagery conditions	BDI-II (SR) (+); Body Sensations Questionnaire (SR) (?); state anxiety (SR) (−); behaviour checklist (SR/CR) (−)	20.5
Vassilopoulos et al. (2012) Greece	Between groups	*n* = 164 Age 10–12, *M* = 10.2 (SD = 0.5)	Positive and negative imagery groups	SASC-R (SR) (+)	Self-imagery questionnaire (SR) (−)	Asked to form a positive or negative image (random allocation)	CDI (SR) (+); Social Events Interpretation Questionnaire (SR) (−)	22
Vassilopoulos and Moberly (2013) Greece	Correlational (and between groups)	*n* = 115 Age 10–12, *M* = 10.2	Benign and negative training groups	SASC-R (SR) (+)	Spontaneous Use of Imagery Scale (SR + CR) (−)	Randomly assigned to benign or negative interpretation training condition	CDI (SR) (+)	20.5

*SR* Self-report, *CR* Clinician rated, *SAD*Social anxiety disorder, *HSA* High socially anxious, *NSA* non-socially anxious, *SPAI-C* Social Phobia and Anxiety Inventory for Children, *SPAI* Social Phobia and Anxiety Inventory, *BFNE* Brief Fear of Negative Evaluation Scale, *FNE* Fear of Negative Evaluation Scale, *QRI-SP* Questionnaire of Recurrent Images in Social Phobia, *STAI* State-Trait Anxiety Inventory, *SASC-R* Social Anxiety Scale for Children-Revised, *PTRS* Perspective-Taking Rating Scale, *BDI-II* Beck Depression Inventory-II, *CDI* Children's Depression Inventory, *DICA* Depression Inventory for Children and Adolescents, *LSAS* Liebowitz Social Anxiety Scale—self-report version, *SPIN* Social Phobia Inventory, *K-SADS-PL* Schedule for Affective Disorders and Schizophrenia for School-Age Children-Present and Lifetime Version, *VVIQ* Vividness of Visual Imagery Questionnaire, *VVQ* Verbalizer-Visualizer Questionnaire, *VISQ* Visual Imagery Style Questionnaire

Psychometric properties: (+) good psychometric properties; (−) poor psychometric properties; (?) unknown psychometric properties

Five studies (Hignett and Cartwright-Hatton 2008; Moriya 2018; Schreiber et al. 2012; Vassilopoulos and Moberly 2013; Vassilopoulos et al. 2012) analysed social anxiety symptoms as a continuum among unselected, community populations. Three studies assigned participants to groups based on self-report measures of social anxiety symptoms (Schreiber et al. 2012; Stopa and Jenkins 2007; Vassilopoulos 2005). Of these three studies, two compared participants with high versus low self-reported social anxiety symptoms (Schreiber et al. 2012; Vassilopoulos 2005), and one study examined different imagery conditions in a high socially anxious group (Stopa and Jenkins 2007). Only two studies included participants meeting diagnostic criteria for SAD group where individuals with SAD were compared to a low (non-clinical) socially anxious group (Ranta et al. 2014; Schreiber and Steil 2013).

The nine studies employed a range of methods to induce and measure imagery. Seven studies induced imagery by instructing participants to hold a particular image in mind; three required participants to hold either a negative or positive self-image (Stopa and Jenkins 2007; Vassilopoulos 2005; Vassilopoulos et al. 2012); two studies instructed participants to imagine an anxiety provoking past experience (Ranta et al. 2014; Schreiber and Steil 2013); one required participants to construct an image of themselves in a described situation (Vassilopoulos and Moberly 2013), and one instructed participants to create an image of their recent performance giving a speech (Hignett and Cartwright-Hatton 2008). The remaining two studies did not induce imagery (Moriya 2018; Schreiber et al. 2012), but instead assessed participants’ general experience of images through questionnaires.

### Associations Between Imagery Characteristics and Social Anxiety (or SAD)

Eight studies reported on the association between imagery characteristics (i.e. imagery perspective, vividness, negative imagery, and image generation ability) and social anxiety symptoms (see Table 2 for summary of results).

**Table 2:** Summary of the results

Citation	Associations between imagery characteristics and social anxiety (or SAD)	Effect of negative imagery for high social anxiety group (or SAD)
Hignett and Cartwright-Hatton (2008)	Increased observer’s perspective associated with higher social anxiety (+) (*d* = 0.37 to 0.61, depending on the anxiety measured used)	
Moriya (2018)	Preference for visual mental imagery and object mental imagery associated with increased social anxiety (+) Preference for spatial mental imagery associated with decreased social anxiety ( +) Vividness of images not associated with social anxiety (−)	
Ranta et al. (2014)	Increased frequency of negative observer’s perspective images for high socially anxious group (high vs. low socially anxious) (+) Increased frequency of negative observer’s perspective images for Social Anxiety Disorder/Sub-clinical Social Anxiety Disorder (SAD vs. no diagnosis) (+)	
Schreiber et al. (2012)	Increased frequency of negative images for high socially anxious group (high vs. low socially anxious) (+)	
Schreiber and Steil (2013)	Increased frequency of negative images for SAD group (SAD vs. control) (+) Increased vividness of negative images for SAD group (SAD vs. control) (+) Increased frequency of observer’s perspective images SAD group (SAD vs. control) (+) (*d* = 0.65)	Increased frequency of negative thoughts for SAD group (SAD vs. control) (+) (*d* = 0.23 to 1.68) Increased frequency of negative emotional reactions for SAD group (SAD vs. control) (+) (*d* = 0.17 to 1.44) Increased anxiety for SAD group (SAD vs. control) (+) (*d* = 1.44)
Stopa and Jenkins (2007)	Increased vividness of negative images for high socially anxious participants in negative imagery condition (negative vs. positive imagery) (−) (*d* = 0.15 to 0.37)	Increased anxiety for high socially anxious participants in negative imagery condition (negative vs. positive imagery) (+) (*d* = 0.1 to 0.91) Poorer self-rated performance for high socially anxious participants in negative imagery condition (negative vs. positive imagery) (+) (*d* = 0.86 to 2.12) Poorer observer-rated performance for high socially anxious participants in negative imagery condition (negative vs. positive imagery) (+) (*d* = 0.27)
Vassilopoulos (2005)	Increased vividness for high socially anxious participants (negative vs. positive imagery) (−) (*d* = 0.33) Increased frequency of negative images for high socially anxious group (high vs. low socially anxious) (+) (*d* = 0.88)	Increased anxiety for high socially anxious participants in negative imagery condition (negative vs. positive imagery) (+) (*d* = 1.36) Increased bodily sensations for high socially anxious participants in negative imagery condition (negative vs. positive imagery) (+) (*d* = 1.27) Increased frequency of bodily sensations for negative compared to positive imagery for high socially anxious group only (high vs. low socially anxious) (+) Poorer self-rated performance for high socially anxious participants in negative imagery condition (negative vs. positive imagery) (+) (*d* = 0.69 to 1.39) Poorer self-rated performance when exposed to negative compared to positive images for high socially anxious group only (high vs. low socially anxious) (+) Increased self-rated anxious appearance and belief that they came across less well when exposed to negative compared to positive images when examining groups combined (−) Poorer observer-rated performance for high socially anxious participants in negative imagery condition (negative vs. positive imagery) (−) (*d* = 0.12–0.32)
Vassilopoulos et al. (2012)		Increased negative interpretations for high socially anxious participants (negative vs. positive imagery) (+) Increased negative interpretations for high socially anxious group in negative imagery condition (high vs. low socially anxious) (+)
Vassilopoulos and Moberly (2013)	Negative self-imagery associated with greater social anxiety (+)	

( +) Significant finding, *p* < 0.05

(−) Non-significant finding, *p* > 0.05

*d* = Cohen’s *d* effect size

#### Imagery Perspective

Three studies investigated the association between imagery perspective and social anxiety symptoms and the findings suggested that observer’s perspective is associated with higher symptom ratings (Hignett and Cartwright-Hatton 2008; Ranta et al. 2014; Schreiber and Steil 2013). In two questionnaire studies that examined the continuous association between social anxiety and imagery perspective, participants were asked to hold a past negative image in mind while reporting on the perspective they had taken. Ranta et al. (2014) found that participants (mean age 15.9 years) self-reporting high symptoms of social anxiety reported significantly more negative observer’s perspective images than participants with low self-reported social anxiety. Furthermore, in Ranta et al. (2014), group comparisons revealed that those participants who met diagnostic criteria for SAD or sub clinical social anxiety (i.e. when all but the functional impairment DSM-IV criterion were met) reported significantly more negative observer’s perspective images than participants without a diagnosis. In another study using clinical populations, Schreiber and Steil (2013) found that young people with a diagnosis of SAD (aged 14–20) reported significantly more frequent observer’s perspective images than non-anxious controls matched for age and gender (*d* = 0.65). However, this effect did not remain significant when depression was controlled in the analysis. Finally, Hignett and Cartwright-Hatton (2008) assigned non-clinical adolescents (age 12–14 and 16–18 years) a three minute speech task which they were told would be rated by a group of peers. After giving the speech, higher social anxiety symptoms, in both age groups, were associated with a higher likelihood to report an observer’s perspective when they were then asked to visualise an image of themselves completing the speech (*d* = 0.37–0.61, depending on the anxiety measure employed).

#### Vividness

Four studies examined the relationship between social anxiety and imagery vividness (i.e. the self-rated degree of richness, amount of detail, and clarity), with mixed findings. No significant associations were found in the three studies that included non-clinical populations, which used questionnaires (Moriya 2018; 18–23 years) and speech tasks (Stopa and Jenkins 2007—mean age 19 years, *d* = 0.15–0.37; Vassilopoulos 2005—mean age 19.7 years, *d* = 0.33). In contrast, in the only study comparing imagery vividness between clinical and non-clinical populations, Schreiber and Steil (2013) found that young people (aged 14–20 years) with SAD reported significantly more vivid negative images than a non-anxious control group when describing a past anxiety provoking social situation.

#### Negative Imagery

Four questionnaire studies explored the relationship between the frequency of negative imagery and social anxiety (or SAD) in young people. When examining the continuous association between social anxiety and frequency of negative images, Vassilopoulos and Moberly (2013; age 10–12 years) found a significant association between greater social anxiety and higher frequency of negative imagery. Furthermore, in studies that have conducted group comparisons, young people with high social anxiety (or SAD) reported significantly greater frequency of negative imagery than low socially anxious (or non-anxious) young people (Schreiber et al. 2012—age 14–20; Schreiber and Steil 2013; Vassilopoulos 2005; mean age 19.7 years, *d* = 0.88). However, notably, while the effect remained significant after controlling for symptoms of depression in the Schreiber and Steil (2013; age 14–20 years) and Schreiber et al. (2012; age 14–20 years) studies, this was not the case in the Vassilopoulos and Moberly (2013) study who used a younger population (aged 10–12 years).

#### Image Generation Ability

One questionnaire study investigated the ability to generate non-emotional mental images in one’s own mind, and this was in a non-clinical population of undergraduates (Moriya 2018; age 18–23 years). A significant association was found between higher social anxiety and greater ability to process visual information about objects and scenes and a lower ability to process information about spatial relations between objects or their parts. The effects of these mental imagery scales on social anxiety were moderated by effortful control (i.e. the ability to control attention and inhibition), suggesting that high effortful control could supress the maladaptive effects of high object mental imagery and low spatial mental imagery on social anxiety.

### Effects of Negative Imagery for Social Anxiety Group (or SAD)

Four studies investigated the effect (in terms of changes in self-reported anxiety, bodily sensations, interpretations, and performance) of manipulating negative imagery on individuals with high levels of social anxiety or SAD.

#### Self-reported Anxiety

Schreiber and Steil (2013) found that young people (14–20 years) with SAD reported experiencing negative self-images with significantly greater distress than non-anxious controls (*d* = 1.44). A similar pattern of results was found in two studies with individuals with high social anxiety, who reported significantly increased state anxiety in a negative compared to a positive imagery condition (Stopa and Jenkins 2007; mean age 19 years, *d* = 0.1 before giving a speech, *d* = 0.91 during a speech; Vassilopoulos 2005; mean age 19.7 years; *d* = 1.36). However, there were no significant differences in self- reported anxiety responses to imagery between the high and low socially anxious groups.

#### Bodily Sensations

Vassilopoulos (2005) found a significant increase in self-reported bodily sensations (e.g. muscle tension and trembling hands) when high (but not low) socially anxious participants (mean age 19.7 years) imagined negative, compared to positive, self-images during a speech task (*d* = 1.27).

#### Performance Ratings

The two studies that examined the effect of negative imagery on self-rated performance appraisals following a speech task both found that high socially anxious participants rated their performance as significantly poorer after holding a negative compared to positive image in mind during a speech task (Stopa and Jenkins 2007—mean age 19 years, *d* = 0.86–2.12; Vassilopoulos 2005—mean age 19.7 years, *d* = 0.69–1.39). Furthermore, in the study of Vassilopoulos (2005), young people with high social anxiety rated their performance as significantly poorer in the negative, compared to positive, imagery condition, and also when compared to the low socially anxious group who did not differ according to the imagery condition.

Findings relating to observer-rated speech performance were less clear, with Stopa and Jenkins (2007) finding that shows significantly poorer observer-rated performance for high socially anxious participants in the negative, compared to positive, imagery condition (*d* = 0.27), but Vassilopoulos (2005) finding that shows no significant difference in observer-rated performance across positive and negative imagery conditions (*d* = 0.12–0.32), or between high and low socially anxious participants.

#### Interpretation of Ambiguous Events

One study investigated the association between imagery and the interpretation of ambiguous situations in the context of social anxiety. Children (age 10–12 years) who reported high levels of social anxiety reported more negative interpretations of an ambiguous situation when instructed to hold a negative, compared to positive, self-image in mind (Vassilopoulos et al. 2012).

### Additional Findings

Schreiber and Steil (2013) found that, when holding a negative image in mind, compared to non-anxious controls, young people (age 14–20 years) with SAD reported significantly higher levels of shame (*d* = 1.19), embarrassment (*d* = 0.91), feeling foolish (*d* = 1.15), insecurity (*d* = 1.12), vulnerability (*d* = 1.00), humiliation (*d* = 1.44), and negative thoughts (*d* = 0.23–1.68).

### Quality of Included Studies

Based on a modified version of Kmet et al. (2004) quality rating checklist, all included studies were rated as ‘medium’ or ‘high’ in their methodological quality. Nonetheless, there was considerable variability in terms of sampling, sample size, the extent to which conclusions could be drawn about the direction of effects, and the methods used to assess imagery.

#### Sampling

Only one study recruited a treatment seeking clinical population (Schreiber and Steil 2013), whereas eight studies recruited from schools and colleges. Two studies included a sample with clinically diagnosed social anxiety disorder (Ranta et al. 2014; Schreiber and Steil 2013) whereas the remaining 7 studies used several different self-report measures to assess social anxiety symptoms. While the reported psychometric properties of these self-report measures indicate that they were reliable and valid, the predominant reliance on non-clinical samples means that caution is needed in drawing conclusions about social anxiety disorder.

#### Sample Size

Sample sizes varied greatly, ranging from 20 (Stopa and Jenkins 2007) to 567 (Schreiber et al. 2012). Only one study reported that they had sufficient power to detect a clinically meaningful effect (Hignett and Cartwright-Hatton 2008); however, most studies did not report their power calculations. Small sample sizes could indicate that studies are underpowered and may result in a Type II error (Columb and Atkinson 2015).

#### Direction of Effects

Five studies did not employ any manipulation of imagery or stress condition in their design (Hignett and Cartwright-Hatton 2008; Moriya 2018; Ranta et al. 2014; Schreiber et al. 2012; Schreiber and Steil 2013), and the cross-sectional designs made it impossible to establish causality. The remaining four studies employed imagery manipulations and used between group designs (Stopa and Jenkins 2007; Vassilopoulos 2005; Vassilopoulos and Moberly 2013; Vassilopoulos et al. 2012) in which participants were assigned to different imagery conditions. The lack of a control group in most of these studies means that it is not possible to determine whether the effects were a direct result of the imagery manipulation, or some other factor that may have also been modified.

#### Assessment of Imagery

Numerous imagery measures were used across the nine studies, including questionnaires and semi-structured interviews. No studies assessed the measures against other measures of imagery, resulting in a lack of concurrent validity, and making it difficult to assess the accuracy or sensitivity of the measures. In addition, the use of retrospective self-report measures of imagery may be biased by socially desirability response and recollection bias. Finally, the validity of using self-report measures to assess highly complex variables, such as negative self-imagery, has been questioned (Schreiber and Steil 2013).

## Discussion

Cognitive models of SAD in adults propose that individuals with SAD experience negative, distorted, observer’s perspective images where they tend to see their worst social fears being realised. The experience of such images is considered to maintain SAD by motivating the individual to make (erroneous) inferences of how they appear to others and engage in safety-seeking behaviours—which prevent belief disconfirmation and ‘contaminate’ social interactions (Clark 2005; Wild et al. 2008).

Our review identified only nine studies that have examined the association between imagery and social anxiety in children and young people. Consistent with the adult models, findings from these studies revealed a significant association (with ‘small’ to ‘large’ effect sizes) between greater frequency of observer’s perspective, rather than field-perspective, imagery and higher social anxiety symptoms (and/or the presence of SAD) in children and young people. Furthermore, there is evidence that the presence of negative imagery among children and young people with social anxiety symptoms and/or SAD is significantly associated with greater self-reported state anxiety, adverse bodily sensations, poorer self-rated performance ratings, and more negative interpretations of ambiguous social events (with ‘small’ to ‘large’ effect sizes). However, it remains unclear whether negative imagery in socially anxious children/young people affects observer-rated performance ratings as findings examining this relationship were inconsistent (with ‘small’ effect sizes). Findings from the adult literature (e.g. Lee and Kwon 2013; Wild et al. 2007) indicate that video feedback and imagery rescripting is an effective therapeutic technique for targeting negative imagery (and its associated effects) in adults with SAD. While this intervention is typically not delivered as part of multi-disorder CBT treatments for child/adolescent SAD (e.g. Kendall and Hedtke 2006), there is preliminary evidence to suggest that similar procedures may also be beneficial for socially anxious adolescents (Parr and Cartwright-Hatton 2009).

### Limitations of the Review

Our findings should be understood in the context of some limitations. First, although we calculated effect sizes where this was possible, some papers lacked sufficient statistical information to inform these calculations, preventing full evaluation of the strength of the existing evidence. Second, this review was limited to studies that had examined features of the most cited and well-established adult cognitive maintenance models of SAD (Clark and Wells 1995; Rapee and Heimberg 1997). It is important to note that several other SAD-specific models that identify negative imagery as a maintenance mechanism have been published recently (e.g. Hofmann 2007; Wong and Rapee 2016). However, although some components of these models are distinct, the role of negative imagery overlaps significantly with the models reviewed here.

In addition to the general lack of studies identified, the included studies also had several important limitations. First, most of the studies included in this review employed samples that were predominantly female, and of the studies that reported ethnicity, all participants identified as Caucasian—thus, limiting the extent to which conclusions can be drawn across different populations. Second, given the lack of studies examining negative imagery in socially anxious children/young people, and given current definitions of adolescence (Sawyer et al. 2018), we elected to include studies with an upper age limit of 24 years—thus, spanning a wide developmental period. Given the broad age ranges included in most studies identified within this review, the extent to which findings are accounted for by developmental factors is uncertain. Indeed, human brain development undergoes vast developmental changes between childhood and adulthood (Supekar et al. 2009) and it remains unclear at what age imagery processes come ‘online’ in children and young people and how their association with affect, and social anxiety specifically, varies with age/development. Future studies would benefit from taking a more developmentally informed approach, with consistency across methods to enable individual patient data meta-analyses that can examine the moderating effects on the associations between imagery and social anxiety going forwards. Third, the most common approach to investigating imagery has been to examine whether increased levels of social anxiety are associated with imagery or (less frequently) compare children with high versus low self-reported social anxiety. Typically, these studies do not assess other anxiety symptoms, and, critically, we identified no studies that have made comparisons between children with SAD and those from other clinical groups—creating significant problems in relating findings, specifically, to social versus other types of anxiety symptoms/disorders. A related issue concerns a failure to consider the influence of comorbidity with other anxiety symptoms/disorders. This is an important omission as there is evidence to suggest moderate to high correlations between social and non-social anxiety symptoms among community populations (Epkins and Heckler 2011) and children with SAD typically meet diagnostic criteria for at least one additional anxiety disorder (Kendall et al. 2010; Waite and Creswell 2014). In addition, with a few notable exceptions (Schreiber et al. 2012; Schreiber and Steil 2013; Vassilopoulos and Moberly 2013), most studies reviewed here have not considered the substantial overlap in, or comorbidity of, social anxiety and depression, meaning that it is also unclear to what extent findings may be ‘driven’ by symptoms of depression. For example, Schreiber and Steil (2013) found that, after controlling for self-reported symptoms of depression, adolescents with SAD reported experiencing negative self-images significantly more frequently, more vividly, and with greater distress than non-anxious controls—but, the relationship between negative observer’s perspective images and SAD did not remain significant. Furthermore, findings from the adult literature suggest that negative imagery also occurs in various other non-social anxiety mental health problems, and imagery rescripting treatment techniques—where the content of negative imagery is directly manipulated—has been employed in the treatment of disorders such as bipolar, snake phobia, and post-traumatic stress disorder (for reviews, see Hackmann and Holmes 2004; Hirsch and Holmes 2007; Holmes et al. 2007). While the first step may be to identify that negative imagery (and its associated effects) is associated with social anxiety symptoms/disorder when compared to low social anxiety or no disorder, it is important to highlight that symptom/disorder-specific associations cannot be concluded from such studies. Third, the literature covered in this review has largely relied on self-report measures to assess imagery. While self-report measures may have several practical advantages (e.g. time- and cost-savings), some of the imagery measures administered consisted of a single item (e.g. Hignett and Cartwright-Hatton 2008) or were developed on the basis of a semi-structured interview about imagery with *adults* (Schreiber et al. 2012). Future research is needed to develop and validate measures that tap into imagery in a way that is effective and appropriate for socially anxious children and adolescents. Finally, more than half of the studies reviewed here examined cross-sectional associations between social anxiety and imagery. Although cross-sectional studies are helpful for establishing the presence of significant associations between childhood/adolescence social anxiety and imagery, they tell us nothing about the direction of effects. For example, the correlation between high social anxiety and greater frequency of negative images (e.g. Vassilopoulos and Moberly 2013) may equally reflect the possibility that negative imagery is a risk for and/or a result of social anxiety. Additionally, the few experimental studies that have been published (Stopa and Jenkins 2007; Vassilopoulos 2005; Vassilopoulos and Moberly 2013; Vassilopoulos et al. 2012) typically lacked a control group and tended to focus on limited aspects of negative imagery, highlighting the need for ongoing work to develop innovative experimental tasks that are appropriate for examining negative imagery in childhood/adolescence social anxiety.

## Conclusions

These limitations notwithstanding the results from this systematic review suggest that there are similarities in the experiences and associated effects of negative imagery across socially anxious adults and socially anxious children/young people. On the basis of the findings of the current review, the front runners as contenders to be treatment targets for social anxiety disorder in children and young people appear to be shifting the focus of attention from observer to field perspective and modifying the imagery so that it is less negative (e.g. using imagery rescripting); notably, these are both treatment targets commonly used in the treatment of adult SAD (Wild et al. 2007). Further studies are now required to establish whether negative imagery and associated effects are specific to childhood/young people with social anxiety, have a causal and/or maintaining role, and potentially to develop treatment interventions that specifically target these psychological mechanisms—ultimately leading to better treatment outcomes for children/young people with social anxiety disorder.

## Electronic Supplementary Material

Below is the link to the electronic supplementary material.

Supplementary material 1 (DOCX 20 kb)
