# Supplementary material for: Mental Imagery in Social Anxiety in Children and Young People: A Systematic Review
Source: Clin Child Fam Psychol Rev. 2020 Apr 15;23(3):379–92. doi: 10.1007/s10567-020-00316-2 (PMC7366604; doi:10.1007/s10567-020-00316-2)
Supplement: Supplementary file 1 — Supplementary material 1 (DOCX 20 kb) [file 10567_2020_316_MOESM1_ESM.docx]

**Supplementary material**

Search Terms

| 1. ((anxiet* OR anxious* OR phobia* OR phobic*) ADJ2 (performance OR social*)) |
| --- |
| 1. (socioanxi* OR sociophobi*). |
| 1. ((blush* OR sweat* OR trembl*) ADJ3 (anxiet* OR anxious* OR chronic* OR excessiv* OR fear* OR severe)) |
| 1. hyperhydrosis OR hyperperspirat* |
| 1. (hyper ADJ (hydrosis OR perspirat*)) |
| 1. ((mute* OR mutism) ADJ2 (elective* OR selective*)) |
| 1. (interpersonal ADJ2 (aversion* OR aversiv* OR confiden* OR difficult* OR disorder* OR distress* OR fear*)) |
| 1. ('inter personal' ADJ2 (aversion* OR aversiv* OR confiden* OR difficult* OR disorder* OR distress* OR fear*)) |
| 1. ((social* OR socio*) ADJ2 (aversion* OR aversiv* OR confiden* OR difficult* OR disorder* OR distress* OR fear*)) |
| 1. ('negative evaluation' ADJ3 (anxiet* OR anxious* OR distress* OR fear*)) |
| 1. (speak* ADJ3 (anxiet* OR anxious* OR distress* OR fear*)) |
| 1. Paruresis |
| 1. (((personalit* OR phobi* OR social* OR socio*) ADJ2 avoid*) OR avoidant disorder) |
| 1. (phobi* ADJ2 neuros*) |
| 1. ((school* ADJ2 (anxiet* OR anxious* OR phobi* OR refuse OR refusal)) OR (shy OR shyness) OR specific phobia*) |
| 1. (phobic disorder*) |
| 1. 1 OR 2 OR 3 OR 4 OR 5 OR 6 OR 7 OR 8 OR 9 OR 10 OR 11 OR 12 OR 13 OR 14 OR 15 OR 16 |
| 1. Imagery |
| 1. representation* |
| 1. ‘observer perspective’ |
| 1. 18 OR 19 OR 20 |
| 1. 17 AND 21 |
